# Plasma concentrations of branched-chain amino acids differ with Holstein genetic strain in pasture-based dairy systems

**DOI:** 10.1038/s41598-021-01564-0

**Published:** 2021-11-17

**Authors:** Ezequiel Jorge-Smeding, Mariana Carriquiry, Gonzalo Cantalapiedra-Hijar, Alejandro Mendoza, Ana Laura Astessiano

**Affiliations:** 1 Departamento de Producción Animal y Pasturas, Facultad de Agronomía, Universidad de la República, Av. Garzón 780, Montevideo, Uruguay; 2 INRAE, Université Clermont Auvergne, VetAgro Sup, UMRH, 63122, Saint-Genès-Champanelle, France; 3 Instituto Nacional de Investigación Agropecuaria. Programa de Producción de Leche. Estación Experimental INIA La Estanzuela, Ruta 50, km 11, 39173 Semillero, Uruguay

**Keywords:** Metabolomics, Physiology

## Abstract

In pasture-based systems, there are nutritional and climatic challenges exacerbated across lactation; thus, dairy cows require an enhanced adaptive capacity compared with cows in confined systems. We aimed to evaluate the effect of lactation stage (21 vs. 180 days in milk, **DIM**) and Holstein genetic strain (North American Holstein, **NAH**, n = 8; New Zealand Holstein, **NZH**, n = 8) on metabolic adaptations of grazing dairy cows through plasma metabolomic profiling and its association with classical metabolites. Although 67 metabolites were affected (*FDR* < 0.05) by DIM, no metabolite was observed to differ between genetic strains while only alanine was affected (*FDR* = 0.02) by the interaction between genetic strain and DIM. However, complementary tools for time-series analysis (ASCA analysis, MEBA ranking) indicated that alanine and the branched-chain amino acids (**BCAA**) differed between genetic strains in a lactation-stage dependent manner. Indeed, NZH cows had lower (*P*-Tukey < 0.05) plasma concentrations of leucine, isoleucine and valine than NAH cows at 21 DIM, probably signaling for greater insulin sensitivity. Metabolic pathway analysis also revealed that, independently of genetic strains, AA metabolism might be structurally involved in homeorhetic changes as 40% (19/46) of metabolic pathways differentially expressed (*FDR* < 0.05) between 21 and 180 DIM belonged to AA metabolism.

## Introduction

Pasture-based dairy systems are gaining scientific interest due to the increasing demand for dairy products associated with lower environmental impact and better animal welfare^1,2^. However, these systems impose restrictive conditions which determine that high genetic merit cows are not able to express their genetic potential for milk yield as their nutritional requirements are not fulfilled due to limited dry matter intake^3^. Furthermore, pasture-based dairy systems are associated with greater nutritional and metabolic challenges for dairy cows because of climatic conditions and variability of pasture allowance and quality along the year^4^. Consequently, adaptive responses of dairy cows are of main importance when they are managed in these systems^5^.

In the dairy cow, at the beginning of lactation, dry matter intake is not sufficient to sustain the high rate of milk synthesis, thus a negative energy balance is established, and adipose and skeletal muscle are mobilized^6,7^. In consequence, it has been widely demonstrated that early lactating cows are characterized by a catabolic state of peripheral tissues associated with the uncoupling of somatotropic axis and a state of insulin resistance^8,9^. In this regard, grazing dairy cows are confronted with more challenging environments than confined dairy cows which determine that these homeorhetic changes are even more dramatic and last for longer during early lactation^10^. However, metabolic adaptations—particularly in early lactation—are affected by the dairy genotype associated with different priorities in nutrients partitioning between milk synthesis vs. body reserves^11,12^. When compared at grazing, NAH (selected for individual milk yields in confined systems) vs. NZH (selected for milk solids and reproductive efficiency in grazing systems) cows had a stronger uncoupling of somatotropic axis and an increased insulin resistance^8,9,13^ which resulted in greater milk yield and a more pronounced loss of body reserves. In addition, differences in homeorhetic strategies between these Holstein strains would also include AA and protein metabolic pathways. Preliminary results obtained by our research group showed greater milk and plasma urea concentrations, as well as lower skeletal muscle mobilization indicated by decreased plasma 3-methyl histidine concentrations, and greater protein oxidative damage for NZH than NAH cows, especially when fed with a strategy that maximized pasture grazing activity^14^.

In parallel, metabolic changes experienced by confined dairy cows during the transition period are increasingly being studied through metabolomic techniques leading to systems biology approaches^15^. Negative associations between plasma short-chain acylcarnitines and non-esterified fatty acids (NEFA), and between large-chain acylcarnitines and insulin suggested that the phospholipidome is involved in the development of insulin resistance^16^. Moreover, dairy cows with high rates of lipid mobilization have been reported to have unique plasma AA profile (i.e.: increased plasma glycine, decreased threonine) in early lactation^16,17^. Recently, Ghaffari et al.^18^ reported that BCAA degradation and methyl-histidine metabolism increased during the transition and suggested these changes could be associated with insulin resistance. However, despite blood metabolomic profiling being successfully used to better understand metabolic adaptations of dairy cows in confined systems^15,19^, there is still no reported literature regarding blood plasma metabolomics studies for pasture-based dairy systems. Therefore, the aim of this work was to study the dynamic of AA metabolism across lactation stages in relationship with milk production and energy metabolism of grazing dairy cows belonging to NAH vs. NZH genetic strains by using a targeted metabolomics approach on blood plasma.

**Table 1:** Least square means, standard error of the mean (SEM) and ANOVA fixed effects of productive variables and metabolite and endocrine concentrations for North American (NAH, n = 8) and New Zealand (NZH, n = 8) Holstein genetic strains (GS) at 21 and 180 days in milk (DIM) on grazing systems.

Parameters^1^	Least square means					*P*-value		
	21 DIM		180 DIM		SEM	GS	DIM	GS $\times$ DIM
	NAH	NZH	NAH	NZH				
FPCM$^2$ (kg/day)	38.9	37.6	29.3	24.5	1.8	0.08	< 0.01	0.21
BW (kg)	556	511	565	528	17	0.03	0.19	0.66
BCS	2.38	2.68	2.48	2.81	0.11	< 0.01	0.06	0.80
NEFA (mmol/L)	0.454	0.535	0.078	0.082	0.057	0.52	< 0.01	0.43
BHB (mmol/L)	0.444	0.404	0.298	0.340	0.044	0.99	0.02	0.32
Urea (mmo/L)	5.60	6.36	7.40	9.14	0.24	< 0.01	< 0.01	0.07
Glucose (mmol/L)	3.07	3.54	2.63	4.38	0.27	< 0.01	0.37	0.01
Insulin (mUI/mL)	5.03	4.63	6.78	8.10	0.55	0.52	< 0.01	0.04

1: Parameters abbreviations: BW: body weight; BCS: body condition score; NEFA: non-esterified fatty acids,

BHB: $$\beta $$-hydroxybutyrate.

FPCM: fat and protein corrected milk, presented as average milk yield of $+/-$ 2.5 days the date in which the blood plasma

samples were collected. FPCM was estimated according to Østergaard et al.^63^ as follows:

FPCM=Milk yield[(0.0383$\times$fat% + 0.0242$\times$protein% + 0.7832)/3.14].

**Table 2:** Fold change and ANOVA fixed effects statistics of plasma metabolites determined by metabolomics and differing between genetic strains (GS; North American Holstein, NAH, n = 8; New Zealand, NZH, n = 8), days in milk (DIM; 21 vs. 180 DIM) or due to the interaction between genetic strains and days in milk.

Compound family	Metabolite	Fold change		GS		DIM		GS $\times$ DIM	
		NZH vs. NAH	21 vs. 180 DIM	*raw-P*$^{1}$	*FDR*$^{2}$	*raw-P*	*FDR*	*raw-P*	*FDR*
$\alpha$-keto acids and derivatives	Pyruvic acid	1.37	0.72	0.50	0.79	0.05	0.10	0.58	0.79
AA	Isoleucine	0.85	0.83	0.01	0.27	0.01	0.04	0.01	0.29
AA	Leucine	0.88	0.78	0.01	0.27	0.01	0.03	0.03	0.30
AA	Valine	0.88	0.77	0.02	0.33	0.01	0.02	0.00	0.21
AA	Glutamic acid	0.81	0.83	0.07	0.58	0.03	0.08	0.09	0.34
AA	Aspartic acid	0.95	0.70	0.09	0.58	< 0.01	< 0.01	0.16	0.46
AA	Asparagine	0.94	0.85	0.22	0.62	0.04	0.08	0.58	0.79
AA	Phenylalanine	1.01	0.76	0.24	0.62	< 0.01	< 0.01	0.37	0.66
AA	Glutamine	0.85	0.78	0.24	0.62	0.02	0.06	0.24	0.58
AA	Lysine	0.92	0.70	0.24	0.62	< 0.01	0.01	0.96	0.98
AA	Threonine	0.99	0.74	0.29	0.62	0.01	0.03	0.06	0.33
AA	Tyrosine	0.97	0.72	0.40	0.74	< 0.01	0.02	0.69	0.87
AA	Cysteine	1.02	0.63	0.58	0.85	< 0.01	< 0.01	0.19	0.51
AA	Methionine	1.02	0.77	0.60	0.85	0.01	0.03	0.38	0.66
AA	Histidine	1.05	0.72	0.73	0.92	< 0.01	< 0.01	0.81	0.92
AA	Glycine	1.09	1.35	0.87	0.98	0.04	0.09	0.18	0.50
AA	Alanine	1.06	0.98	0.91	0.98	0.50	0.66	< 0.01	0.02
AA	Tryptophan	1.08	0.70	0.93	0.98	< 0.01	< 0.01	0.78	0.91
AA related	Oxoproline	0.89	0.94	< 0.01	0.22	0.04	0.09	0.43	0.70
AA related	Pipecolinic acid	0.64	0.70	0.01	0.27	0.04	0.09	0.11	0.38
AA related	Homocystine	0.94	1.03	0.03	0.49	0.97	0.98	0.46	0.72
AA related	5-Hydroxynorvaline	0.90	0.81	0.04	0.51	< 0.01	0.01	0.75	0.90
AA related	$\alpha$-aminoadipic acid	0.92	0.70	0.05	0.51	< 0.01	< 0.01	0.03	0.30
AA related	N-acetylglycine	0.82	2.15	0.06	0.58	< 0.01	< 0.01	0.36	0.66
AA related	2-Ketoisocaproic acid	0.85	1.05	0.08	0.58	0.92	0.97	0.08	0.33
AA related	Creatinine	1.01	1.01	0.09	0.58	0.38	0.54	0.03	0.30
AA related	3-Hydroxy-3-methylglutaric acid	0.86	0.78	0.10	0.58	0.07	0.14	0.12	0.38
AA related	Ornithine	0.94	0.57	0.14	0.58	< 0.01	0.00	0.54	0.78
AA related	Citrulline	0.96	0.83	0.14	0.58	0.01	0.03	0.55	0.78
AA related	3-Aminoisobutyric acid	0.62	2.23	0.15	0.58	< 0.01	0.01	0.01	0.24
AA related	Phenaceturic acid	0.95	0.75	0.15	0.58	< 0.01	0.02	0.50	0.76
AA related	Kynurenine	1.30	0.55	0.19	0.58	< 0.01	< 0.01	0.50	0.76
AA related	Guanidinosuccinate	1.19	0.73	0.27	0.62	0.00	0.01	0.35	0.66
AA related	Allantoic acid	1.18	1.25	0.31	0.64	0.02	0.04	0.11	0.36
AA related	Cystine	1.19	0.64	0.37	0.71	< 0.01	< 0.01	0.45	0.72
AA related	5-Aminovaleric acid	0.99	1.42	0.40	0.74	0.04	0.10	0.04	0.30
AA related	Aminomalonate	1.06	1.30	0.57	0.85	0.04	0.09	0.21	0.55
AA related	3-(4-Hydroxyphenyl)propionic acid	1.10	0.82	0.73	0.92	< 0.01	0.02	0.35	0.66
AA related	Trans-4-hydroxyproline	1.11	1.46	0.91	0.98	< 0.01	0.01	0.35	0.66
AA related	Phenylacetic acid	1.09	0.76	0.94	0.98	< 0.01	0.01	0.28	0.60
AA related	Cystathionine	1.08	0.89	0.96	0.98	0.01	0.02	0.10	0.35
Bile acids	Cholic acid	1.20	3.00	0.31	0.64	< 0.01	0.01	0.04	0.31
Bile acids	Deoxycholic acid	1.17	2.69	0.34	0.67	0.01	0.02	0.07	0.33
Biogenic amines	Phenylethylamine	1.68	0.23	0.25	0.62	< 0.01	< 0.01	0.01	0.24
Carbohydrates and related	Xylose	0.92	1.20	0.00	0.22	0.16	0.28	0.94	0.97
Carbohydrates and related	Erythritol	1.21	1.06	0.01	0.22	0.90	0.97	0.30	0.62
Carbohydrates and related	1,5-Anhydroglucitol	0.89	1.59	0.06	0.58	0.02	0.04	0.32	0.65
Carbohydrates and related	Xylulose	0.73	1.33	0.08	0.58	0.17	0.30	0.42	0.70
Carbohydrates and related	Mannose	1.03	0.86	0.23	0.62	< 0.01	0.01	0.26	0.59
Carbohydrates and related	Glycerol-$\alpha$-phosphate	1.39	0.66	0.28	0.62	0.01	0.04	0.04	0.31
Carbohydrates and related	$\alpha$-ketoglutarate	0.91	0.55	0.30	0.63	< 0.01	0.01	1.00	1.00
Carbohydrates and related	Gluconic acid	0.91	0.64	0.63	0.87	0.01	0.03	0.72	0.88
Carbohydrates and related	Glucose	1.13	0.89	0.73	0.92	0.01	0.02	0.22	0.55
Carbohydrates and related	Galactonic acid	0.98	0.59	0.75	0.92	0.01	0.03	0.54	0.78
Carboxylic acids	Isocitric acid	0.93	1.23	0.11	0.58	0.06	0.12	0.16	0.48
Carboxylic acids	Aconitic acid	0.95	1.35	0.15	0.58	0.01	0.04	0.49	0.76
Carboxylic acids	Citric acid	0.94	1.29	0.16	0.58	0.02	0.04	0.05	0.33
Carboxylic acids	Salicylic acid	1.08	0.73	0.96	0.98	< 0.01	0.01	0.63	0.84
Cresols and related compounds	p-tolyl glucuronide	0.61	0.59	0.05	0.51	< 0.01	< 0.01	0.00	0.21
Cresols and related compounds	p-cresol	0.93	0.56	0.19	0.58	0.00	0.01	0.33	0.65
Fatty acids	Arachidic acid	0.96	1.02	0.00	0.22	0.71	0.82	0.08	0.33
Fatty acids	Stearic acid	0.99	1.30	0.04	0.51	0.03	0.07	0.05	0.33
Fatty acids	Nonadecanoic acid	1.04	1.49	0.14	0.58	0.02	0.04	0.08	0.33
Fatty acids	Linoleic acid	0.98	1.44	0.14	0.58	0.01	0.03	0.12	0.38
Fatty acids	Myristic acid	1.05	2.01	0.41	0.74	< 0.01	0.01	0.06	0.33
Fatty acids	Heptadecanoic acid	1.08	1.95	0.44	0.75	< 0.01	0.01	0.07	0.33
Fatty acids	Palmitic acid	1.05	1.40	0.47	0.76	< 0.01	0.02	0.07	0.33
Fatty acids	Arachidonic acid	1.07	1.73	0.65	0.87	< 0.01	0.02	0.22	0.55
Fatty acids	Isolinoleic acid	1.13	0.77	0.78	0.93	0.04	0.09	0.98	0.99
Fatty acids	Palmitoleic acid	1.23	3.99	0.85	0.97	< 0.01	< 0.01	0.06	0.33
Fatty acids	9-Myristoleate	1.07	1.61	0.87	0.98	0.01	0.03	0.03	0.30
Fatty acids	Cerotinic acid	1.04	0.69	0.98	1.00	0.01	0.03	0.29	0.61
Glycerides	1-Monopalmitin	1.18	0.75	0.25	0.62	< 0.01	< 0.01	0.26	0.58
Glycerides	1-Monostearin	1.08	0.73	0.96	0.98	< 0.01	< 0.01	0.37	0.66
Hydroxy acids and derivatives	2-Hydroxyglutaric acid	0.95	1.45	0.32	0.66	< 0.01	0.01	0.54	0.78
Hydroxy acids and derivatives	2-Hydroxybutanoic acid	0.94	1.89	0.45	0.75	< 0.01	0.02	0.28	0.60
Hydroxy acids and derivatives	4-Hydroxybutyric acid	1.05	1.40	0.70	0.92	< 0.01	0.02	0.85	0.96
Imides	Maleimide	1.27	0.81	0.17	0.58	0.04	0.09	0.09	0.34
Indoles and derivatives	Indole-3-propionic acid	1.57	0.86	0.09	0.58	0.02	0.06	0.74	0.90
Indoles and derivatives	Indoxyl sulfate	1.05	0.59	0.88	0.98	< 0.01	< 0.01	0.93	0.97
Inorganic compounds	Phosphate	1.17	0.85	0.43	0.75	0.01	0.03	0.01	0.29
Keto-acids	2-Ketobutyric acid	1.00	0.70	0.59	0.85	0.02	0.04	0.21	0.55
Nitrogenous bases and related	Uric acid	0.77	1.58	0.01	0.28	0.01	0.02	0.38	0.66
Nucleoside and nucleotide analogues	Pseudo uridine	1.10	1.69	0.94	0.98	< 0.01	0.01	0.44	0.70
Phenol esters	4-Hydroxyphenylacetic acid	1.34	1.79	0.17	0.58	< 0.01	0.01	0.53	0.78
Quinolones and derivatives	2,8-Dihydroxyquinoline	0.97	1.47	0.26	0.62	< 0.01	0.01	0.57	0.79
Sterols	Cholesterol	1.31	0.89	0.05	0.51	0.09	0.17	0.88	0.97
Sugar alcohol	Xylitol	0.95	1.32	0.07	0.58	0.02	0.06	0.14	0.42
Sugar alcohol	Isothreitol	1.06	1.32	0.49	0.79	< 0.01	< 0.01	0.64	0.85
Sugar alcohol	Glycerol	1.06	1.54	0.60	0.85	< 0.01	0.02	0.39	0.66
Terpenoids and derivatives	Phytanic acid	1.14	0.70	0.44	0.75	< 0.01	0.02	0.59	0.80
Vitamins and cofactors	$\alpha$-tocopherol	1.36	0.75	0.03	0.49	0.01	0.04	0.24	0.58

1: *raw-P* value according to ANOVA; 2: *P*-value adjusted by false discovery rate.

**Table 3:** Metabolic pathways differentially expressed accross lactation stages (21 vs. 180 days in milk; DIM) or by genetic strains (North American Holstein vs. New Zealand Holstein; NAH vs. NAZ) at 21 days in milk. Total compounds, hits and pathway impacts are presented along with P-statistics.

	Metabolic pathways	Total comp^1^	Hits$^2$	*raw-P*$^3$	*FDR*$^3$	Impact$^4$
**a. 21 vs. 180 DIM**						
AA and protein	Phenylalanine metabolism	12	6	4.3E-08	7.6E-07	0.60
AA and protein	Arginine biosynthesis	14	8	4.6E-08	7.6E-07	0.41
AA and protein	Arginine and proline metabolism	38	6	5.2E-08	7.6E-07	0.36
AA and protein	Tyrosine metabolism	42	4	1.1E-07	1.3E-06	0.16
AA and protein	Histidine metabolism	16	3	3.1E-07	3.0E-06	0.22
AA and protein	Alanine, aspartate and glutamate metabolism	28	11	8.7E-07	7.2E-06	0.67
AA and protein	$\beta$-Alanine metabolism	21	5	1.3E-06	9.1E-06	0.50
AA and protein	Glycine, serine and threonine metabolism	34	8	3.9E-06	1.9E-05	0.52
AA and protein	Cysteine and methionine metabolism	33	8	4.6E-06	1.9E-05	0.50
AA and protein	Tryptophan metabolism	41	4	4.9E-06	1.9E-05	0.25
AA and protein	Lysine degradation	25	3	5.6E-06	2.0E-05	0.14
AA and protein	Phenylalanine, tyrosine and tryptophan biosynthesis	4	2	9.3E-06	2.8E-05	1.00
AA and protein	Aminoacyl-tRNA biosynthesis	48	19	9.5E-06	2.8E-05	0.17
AA and protein	Taurine and hypotaurine metabolism	8	1	2.0E-05	5.0E-05	0.00
AA and protein	Thiamine metabolism	7	1	2.0E-05	5.0E-05	0.00
AA and protein	D-Glutamine and D-glutamate metabolism	5	3	4.4E-05	9.2E-05	1.00
AA and protein	Valine, leucine and isoleucine biosynthesis	8	6	9.2E-04	1.5E-03	0.00
AA and protein	Valine, leucine and isoleucine degradation	40	4	6.4E-03	9.3E-03	0.01
AA and protein	Nitrogen metabolism	6	2	6.6E-03	9.4E-03	0.00
Carbohydrates	Glyoxylate and dicarboxylate metabolism	32	9	4.5E-06	1.9E-05	0.28
Carbohydrates	Fructose and mannose metabolism	20	2	1.3E-04	2.4E-04	0.03
Carbohydrates	Galactose metabolism	27	10	1.7E-04	3.1E-04	0.29
Carbohydrates	Pentose and glucuronate interconversions	18	6	4.0E-04	7.0E-04	0.50
Carbohydrates	Amino sugar and nucleotide sugar metabolism	37	5	5.0E-03	7.5E-03	0.11
Energy central	Citrate cycle (TCA cycle)	20	7	8.4E-06	2.7E-05	0.35
Energy central	Propanoate metabolism	23	4	3.8E-05	8.1E-05	0.04
Energy central	Butanoate metabolism	15	6	9.8E-05	1.9E-04	0.03
Energy central	Ubiquinone and other terpenoid-quinone biosynthesis	9	1	4.2E-04	7.2E-04	0.00
Energy central	Biotin metabolism	10	1	7.4E-04	1.2E-03	0.00
Energy central	Glycolysis / Gluconeogenesis	26	1	1.7E-02	2.4E-02	0.10
Energy central	Synthesis and degradation of ketone bodies	5	1	2.1E-02	2.9E-02	0.00
Energy central	Pentose phosphate pathway	22	3	3.2E-02	4.1E-02	0.05
Heme biosynthesis	Porphyrin and chlorophyll metabolism	30	2	2.3E-03	3.5E-03	0.00
Lipid	Fatty acid biosynthesis	47	4	2.5E-06	1.6E-05	0.01
Lipid	Arachidonic acid metabolism	37	1	3.1E-06	1.8E-05	0.32
Lipid	Fatty acid elongation	39	1	4.1E-06	1.9E-05	0.00
Lipid	Linoleic acid metabolism	5	1	1.4E-05	3.9E-05	1.00
Lipid	Biosynthesis of unsaturated fatty acids	36	6	2.1E-05	5.0E-05	0.00
Lipid	Fatty acid degradation	39	2	2.2E-05	5.2E-05	0.00
Lipid	Primary bile acid biosynthesis	46	3	4.8E-05	9.5E-05	0.06
Lipid	Glycerolipid metabolism	16	2	1.5E-03	2.4E-03	0.33
Lipid	Inositol phosphate metabolism	30	4	3.3E-02	4.2E-02	0.16
Nitrogenous bases	Purine metabolism	66	4	3.3E-05	7.3E-05	0.02
Redox	Glutathione metabolism	28	5	1.0E-08	5.8E-07	0.12
Redox	Ascorbate and aldarate metabolism	10	4	2.2E-02	2.9E-02	0.50
Vitamins and coenzymes	Pantothenate and CoA biosynthesis	19	7	6.0E-06	2.0E-05	0.06
Lipid	Steroid hormone biosynthesis	75	1	5.8E-02	7.2E-02	0.01
Anitbiotic biosynthesis	Neomycin, kanamycin and gentamicin biosynthesis	2	2	6.9E-02	8.3E-02	0.00
**b. NAH vs. NZH cows at early lactation (21 DIM)**						
AA and protein	Valine, leucine and isoleucine degradation	40	4	6.8E-04	2.2E-02	0.01
AA and protein	Valine, leucine and isoleucine biosynthesis	8	6	7.7E-04	2.2E-02	0.00
AA and protein	Lysine degradation	25	3	1.2E-03	2.2E-02	0.14
Redox	Selenocompound metabolism	20	1	3.8E-03	5.5E-02	0.00
AA and protein	Aminoacyl-tRNA biosynthesis	48	19	6.0E-03	6.9E-02	0.17

1: Total metabolites theoretically considered by the *Bos taurus* KEGG database for the current metabolic pathways.

2: Metabolites efectively quantified in the current study and belonging to the identified pathway.

3: *Raw-P* and *FDR*-adjusted P values obtained with the Global Test.

4: Topological analysis of impact of the current metabolic pathway.

## Results

### Productive performance and plasma metabolite and endocrine profile

The fat and protein-corrected milk yield tended to be greater (*P* = 0.08) for NAH than NZH cows (34.1 vs. $31.1 \pm 1.8$ kg/day) and decreased (*P* < 0.01) from 21 to 180 DIM (Table 1). Cow body weight (**BW**) was greater (*P* = 0.03) and body condition score (**BCS**) was lower (*P* < 0.01) for NAH than NZH. In addition, BCS tended to increase (*P* = 0.06) from 21 to 180 DIM for both, NAH and NZH cows. Plasma concentrations of NEFA and BHB were greater (*P* $\le$ 0.02) at 21 than 180 DIM with no differences between the genetic strains, while urea concentrations were lower (*P* < 0.01) for NAH than NZH cows and increased (*P* < 0.01) for all cows from 21 to 180 DIM. Both glucose and insulin were affected (both *P* $\le$ 0.04) by the interaction between genetic strain and DIM as glucose concentration increased (*P* < 0.01) at 180 DIM only in NZH cows, while insulin increased (*P* < 0.01) for both strains but NZH reached greater (*P* = 0.04) concentrations than NAH cows at this time.

**Figure 1:**
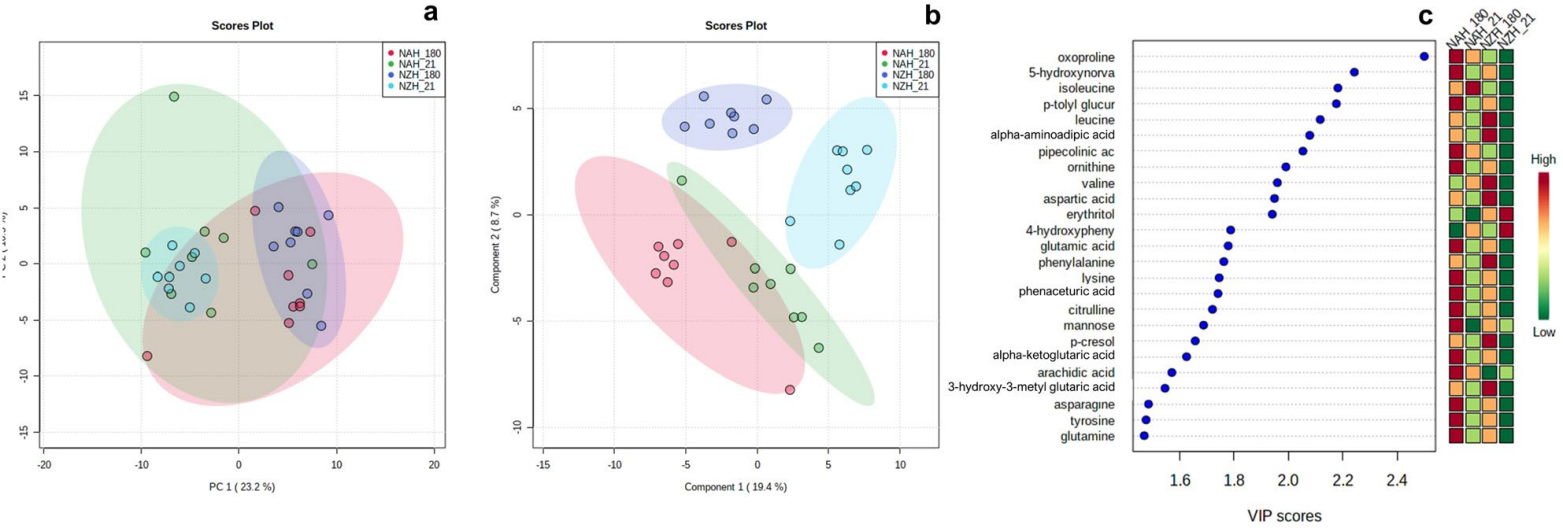
Scores plot for PCA (**a**), PLS-DA (**b**) of North American (NAH, n = 8) and New Zealand (NZH, n =8) Holstein cows under grazing conditions at 21 (green and sky-blue dots, respectively) and 180 (red and purple dots, respectively) days in milk. The VIP scores plot (**c**) is based on the top 25 metabolites with the highest VIP values for the 1st component of PLS-DA. Green to red color denote low to high plasma concentrations of the current metabolite.

**Figure 2:**
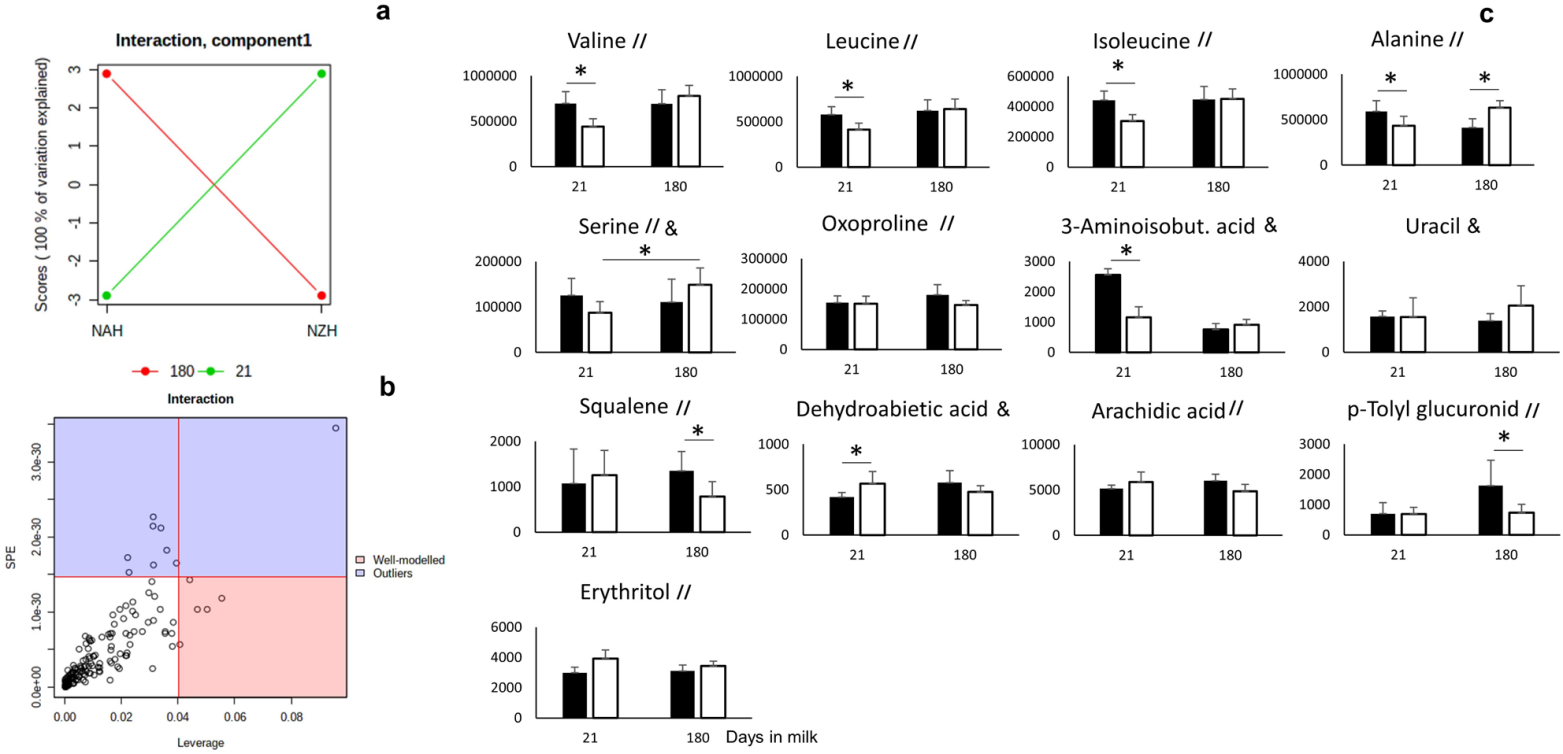
Genetic strain (North American Holstein, NAH, n = 8; New Zealand Holstein, NZH, n = 8) and days in milk interaction effect plots based on 1st component of ASCA (**a**), metabolites well modeled for ASCA interaction model (**b**) and bar plot of metabolites identified to have different temporal patterns between NAH and NZH cows according to ASCA (denoted by&) and/or top 10 MEBA’s ranking (denoted by //). Significant differences between means according to Tukey test are denoted by * whenever the ANOVA interaction effect *P*-value $\le$ 0.10. The x-axis denote days in milk, the y-axis denote ion intensity peak height and error bars indicate standard deviation.

**Figure 3:**
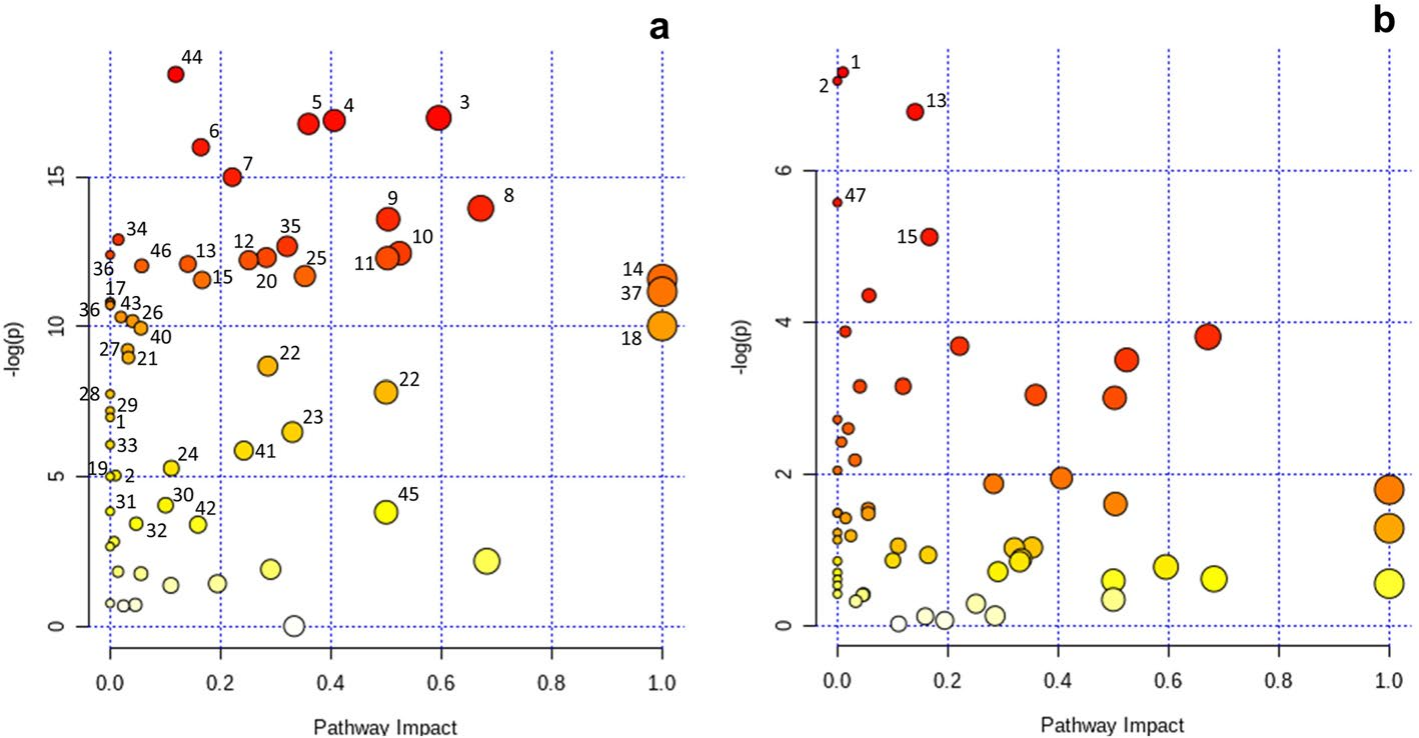
Metabolic pathway análisis based on metabolic enrichment analysis and topologic analysis of metabolomic profiles comparing metabolic pathways shifts when comparing: (**a**) 21 vs. 180 days in milk, or (**b**) North American vs. New Zealand Holstein at 21 days in milk. Increasingly red colors indicate lower *P*-values (more significant shift in the pathway), greater circle size indicate greater pathway impact. Numerous depict pathways with significant regulation shift: 1: Branched-chain amino acids biosynthesis, 2: branched-chain amino acids degradation, 3: phenylalanine metabolism, 4: arginine biosynthesis, 5: arginine and proline metabolism, 6: tyrosine metabolism, 7: histidine metabolism, 8: alanine, aspartate and glutamate metabolism, 9: $\beta$-Alanine metabolism, 10: glycine, serine and threonine metabolism, 11: cysteine and methionine metabolism, 12:tryptophan metabolism, 13: lysine degradation, 14: phenylalanine, tyrosine and tryptophan biosynthesis, 15: aminoacyl-tRNA biosynthesis, 16: taurine and hypotaurine metabolism, 17: thiamine metabolism, 18: glutamine and glutamate metabolism, 19: nitrogen metabolism, 20: glyoxylate and dicarboxylate metabolism, 21: fructose and mannose metabolism, 22: galactose metabolism, 23: pentose and glucuronate interconversions, 24: amino sugar and nucleotide sugar metabolism, 25: citrate cycle, 26: propanoate metabolism, 27: butanoate metabolism, 28: ubiquinone and other terpenoid-quinone biosynthesis, 29: biotin metabolism, 30: glycolysis/gluconeogénesis, 31: synthesis and degradation of ketone bodies, 32: pentose phosphate pathway, 33: porphyrin and chlorophyll metabolism, 34: fatty acid biosynthesis, 35: arachidonic acid metabolism, 36: fatty acid elongation, 37: linoleic acid metabolism, 38: biosynthesis of unsaturated fatty acids, 39: fatty acid degradation, 40: primary bile acid biosynthesis, 41: glycerolipid metabolism, 42: inositol phosphate metabolism, 42: purine metabolism, 43: glutathione metabolism, 44: ascorbate and aldarate metabolism, 45: pantothenate and CoA biosynthesis, 46: hisitidine metabolism, 47: selenocompound metabolism.

**Figure 4:**
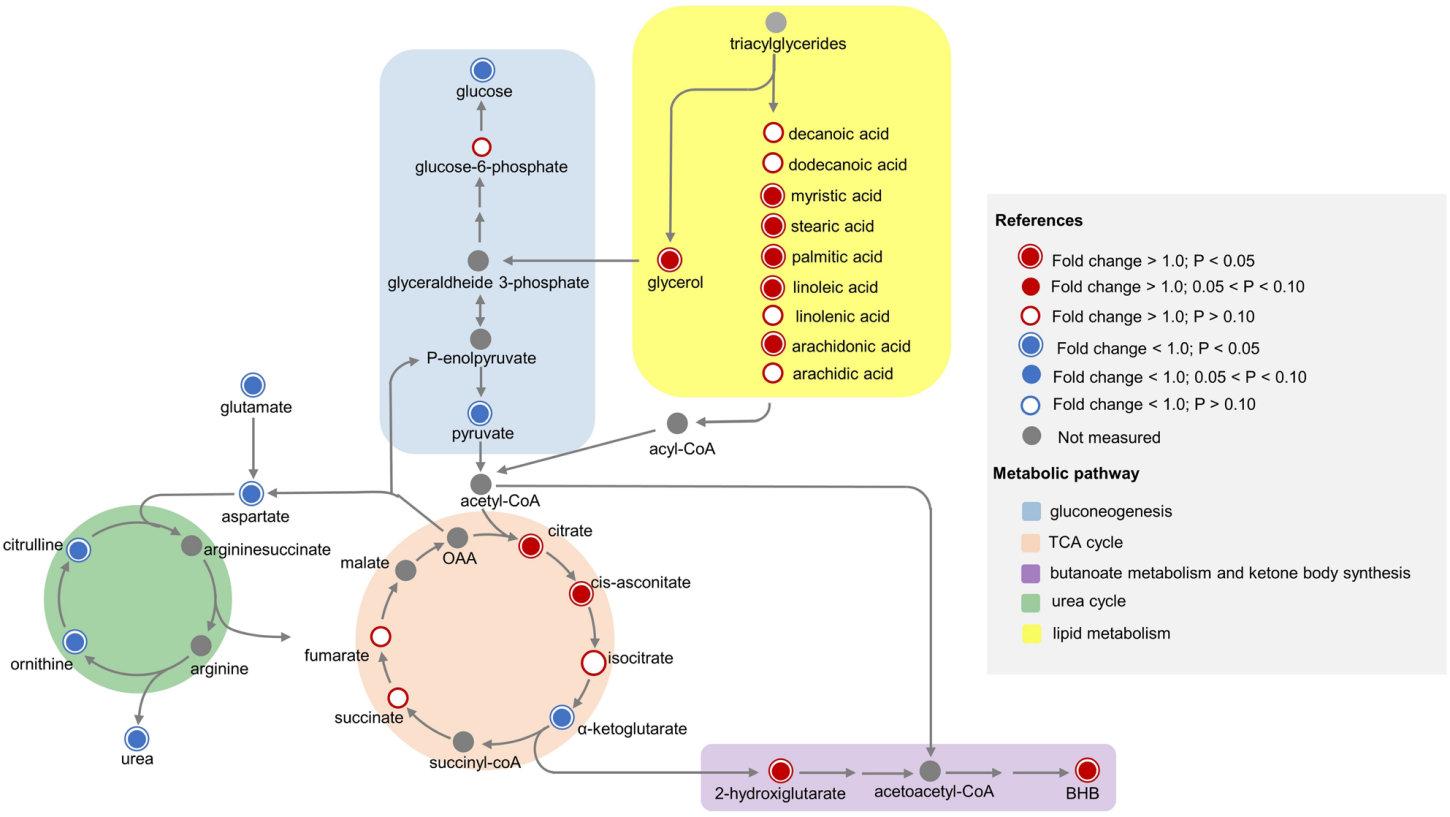
Main metabolic pathways identified to be differentially regulated at early vs. mid lactation stage (21 vs. 180 days in milk) according to metabolic pathways analysis performed on blood plasma metabolomic data of multiparous Holstein cows under grazing conditions. Colored circles depict effectively measured compound, while gray circles correspond to undetected ones. Negative and positive fold changes for each metabolite when comparing 21 vs. 180 days in milk are denoted by blue or red circles, respectively. Color background group metabolic pathways indicated in the figure legend, as well as significance levels.

**Figure 5:**
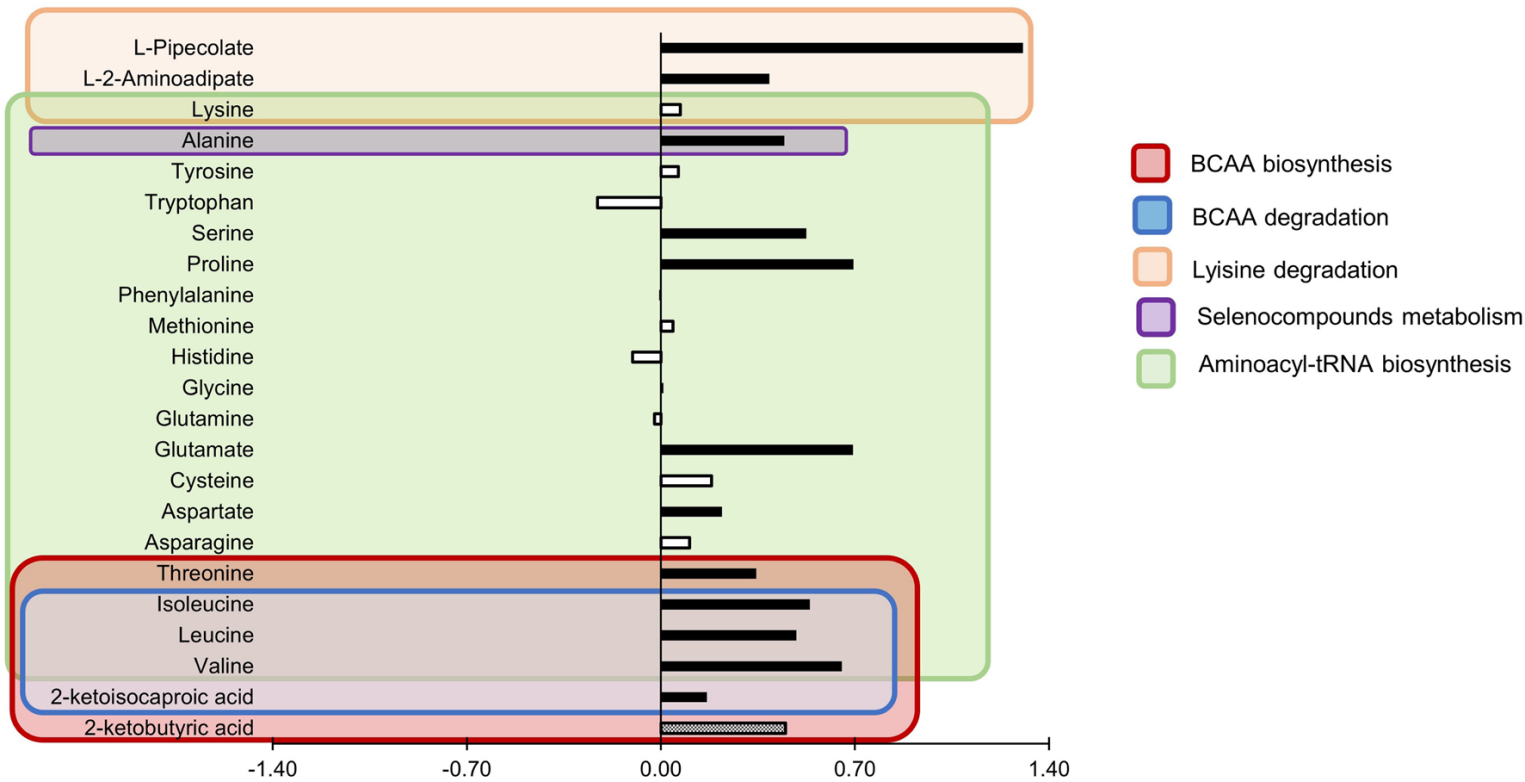
Log 2-fold change of quantified metabolites belonging to metabolic pathways identified to be differentially regulated (FDR < 0.10) between genetic strains (North American Holstein, NAH, n = 8; New Zealand Holstein, NZH, n = 8) NAH at 21 days in milk. Positive values mean greater concentrations in NAH relative to NZH cows. Black bars indicate *P* $\le$ 0.05, gray bars are metabolites with 0.05 < *P* $\le$ 0.10 and white bars indicate *P* > 0.10. Background colors indicate metabolic pathways indicated in the figure legend.

**Figure 6:**
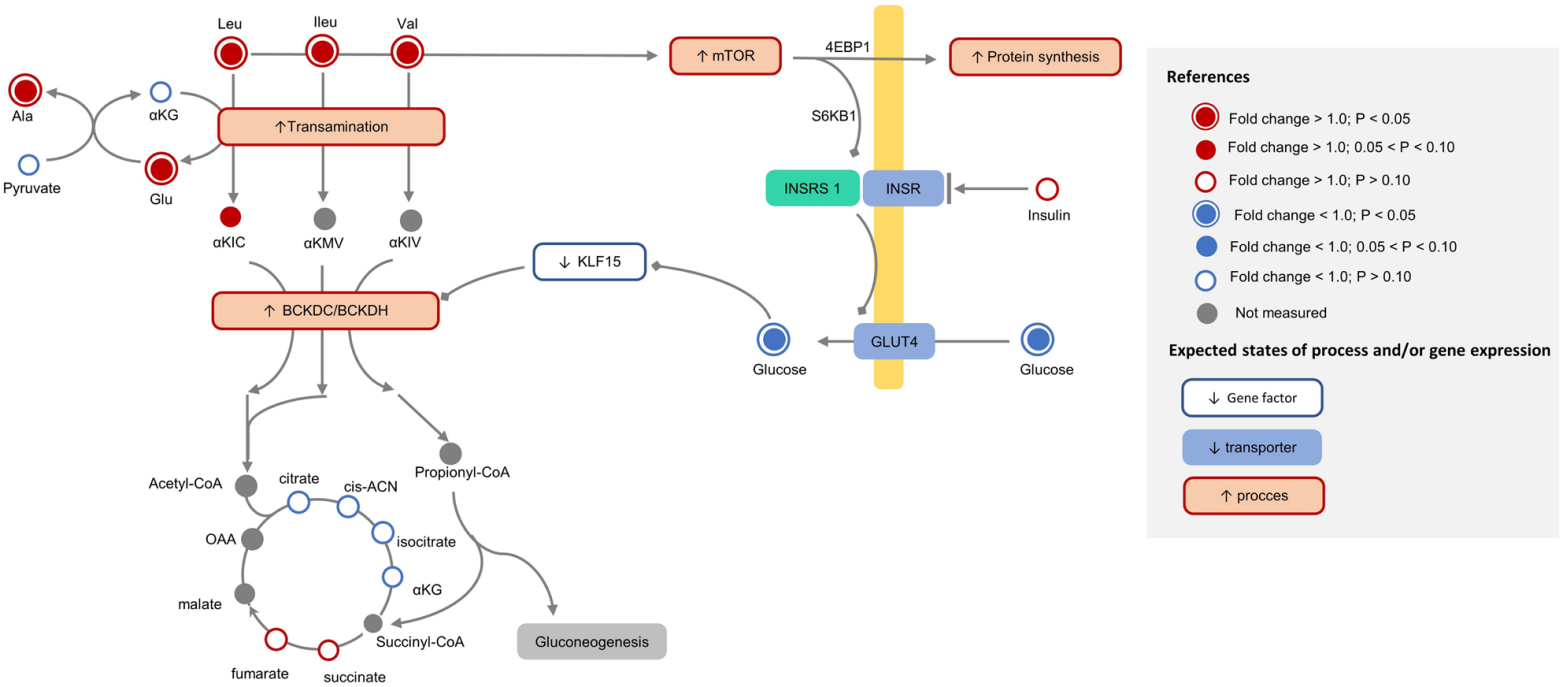
Integrative metabolic interpretation map of branched-chain amino acids (leucine, isoleucine, valine) metabolism and insulin sensitivity differences between genetic strains (North American Holstein, NAH, n = 8; New Zealand Holstein, NZH, n = 8) at early lactation stage (21 days in milk) under grazing conditions. Red-colored circles indicate greater, while blue circles lower plasma concentrations of metabolites for NAH vs. NZH cows. Color code for significance levels is stated in the figure legend. Colored-rectangles indicate the expected regulation state of process or transport/gene expression as stated in the figure legend.

### Metabolomic profiling and cluster analysis

The final metabolomic dataset consisted of 172, out of 200 metabolites assayed by the GCToF/MS method, which were effectively identified above the limit of detection (i.e. metabolite’s identifier ion with peak intensity greater than the limit of detection in at least 80 % of the plasma samples) (Supplementary Table S2 online). Days in milk had a stronger effect than the genetic strain on principal component analysis (**PCA**) clustering (Fig. 1a). In addition, PCA showed that it was easier to discriminate early vs. mid-lactating NZH than NAH cows. Principal components 1 and 2 accounted for 33.7 % of the variation. The partial least square discriminant analysis (**PLS-DA**) confirmed that the animals could be better discriminated by stage of lactation for NZH than NAH cows (Fig. 1b). The best classification PLS-DA model, based on the first 3 components, had an acceptable predictive ability $Q^2$ ($R^2$= 0.94, $Q^2$ = 0.54). Fifty-nine, out of the 172 metabolites, had a value in importance projection (**VIP**) > 1.0. Among them, seven metabolites including oxoproline, 5-hydroxynorvaline, isoleucine, p-tolyl glucuronide, leucine, $\alpha$-aminoadipic acid and L-pipecolate had a VIP > 2.0. For all of these metabolites , the lowest concentration was observed for NZH at 21 DIM (Fig. 1c).

### Univariate analysis and correlations

According to ANOVA analysis, 67 of the quantified metabolites varied with DIM (*FDR* < 0.05; Table 2) whereas there was no metabolite affected by genetic strain after *P*-value adjustment by FDR. Amino acids (n = 12) or AA-related compounds (n =15) represented 40% of the metabolites differing between stages of lactation, most of them (23/27) being associated with lower concentrations for 21 vs. 180 DIM (relative peak height -25 % on average, *FDR* < 0.05). A few AA-related compounds (e.g.: N-acetylglycine, 3-aminoisobutyric acid, trans-4-hydroxyproline, and allantoic acid) had a greater concentration (*FDR* $\le$ 0.05) for 21 vs. 180 DIM (Table 2). In addition, almost all the fatty acids (8/9) were affected by DIM as well as all the hydroxy acids and derivatives (3/3), sugar alcohols (2/2), and bile acids (1/2) which had greater concentrations (*FDR* $\le$ 0.05) for 21 vs. 180 DIM. Conversely, glycerides (2/2), and carbohydrates and related compounds (6/7) had lower concentrations at 21 vs. 180 DIM (Table 2). Despite none of the quantified metabolites having an *FDR* $\le$ 0.05 when comparing genetic strains, 16 metabolites had a *raw-P* $\le$ 0.05, most of them being AA or AA-related compounds (n = 12) and fatty acids (n = 2) with greater concentrations for NAH than NZH cows (Table 2).

Additionally, only alanine was affected (*FDR* = 0.02) by the interaction between genetic strain and DIM as it was greater at 21 DIM but lower at 180 DIM for NAH vs. NZH dairy cows (Table 2, Fig. 2c). However, changing patterns of metabolites between genetic strains in a time-dependent manner were also assessed by both, *ANOVA simultaneous component analysis* (**ASCA**) and the *Bayes statistical time-series analysis* (**MEBA**) ranking. According to the ASCA analysis, a valid model was obtained for the interaction effect (genetic strain $\times$ DIM, *P* = 0.04, 4/100 permutations) (Supplementary Fig. 1 online). Four metabolites were well modelled (leverage > 0.040, SPE < 1.5 $\times$ 10$^{-30}$) by this interaction model (Fig. 2b,c). Among these 4 metabolites, dehydroabietic acid was decreased and 3-aminoisobutyric acid was increased for NAH vs. NZH cows only at 21 DIM (Fig. 2c). In addition, serine increased from 21 to 180 DIM only for NZH cows, while uracil was lower for NAH than NZH cows at 180 DIM but no significant differences were detected after Tukey’s test for multiple comparisons (Fig. 2c). In addition, the top-10 metabolites (MEBA ranking top-10, Hotelling’s $\mathrm{T}^{2} > 10.0$) with the greatest differences in temporal profiles between genetic strains were alanine, BCAA (valine, isoleucine, leucine), arachidic acid, oxoproline, squalene, p-tolyl glucuronide, erythritol and serine (Supplementary Table S3 online). Specifically, concentrations of BCAA were greater for NAH than NZH cows only at 21 DIM (Fig. 2c). In addition, p-tolyl glucuronide increased from 21 to 180 DIM for NAH cows reaching greater concentrations than their NZH counterparts (Fig. 2c). Except for uracil (*raw-P* = 0.054), arachidic acid (*raw-P* = 0.08), erythritol , and oxoproline (*P* > 0.10), all of these metabolites identified by both ASCA and/or MEBA ranking had a *raw-P* $\le$ 0.05 for the interaction effect (genetic strain $\times$ DIM) according to ANOVA results.

### Metabolic pathways analysis across lactation stages

This analysis revealed that 46 metabolic pathways differed (*FDR* $\le$ 0.05) when comparing early vs. mid-lactation stages (21 vs. 180 DIM) independently of genetic strain. Most of them (19/46) corresponded to AA and protein metabolism including BCAA and essential AA metabolism, glutamine and glutamate metabolism, among others (Table 3, Fig. 3). Except for a few metabolites (e.g.: glycine, glycerate, 4-hydroxiphenylacetate, indole-3-acetate, $\beta$-alanine and 3-ureidopropionate), these pathways were associated with lower concentrations at 21 vs. 180 DIM (Fig. 4; Supl. Figure 2). In addition, some pathways involved in carbohydrates metabolism likewise glyoxylate and dicarboxylate metabolism also differed (*FDR* < 0.05) between lactation stages. Central energy pathways included citric acid cycle (**TCA**), butanoate metabolism, ketone bodies synthesis and degradation, among others (Table 3, Figs. 3 and 4). It was related to some metabolites such as citrate and cis-aconitate that were increased (*FDR* < 0.05) at 21 vs. 180 DIM, while others such as $\alpha$-ketoglutarate and $\alpha$-ketobutyric acid were decreased (*FDR* $\le$ 0.04) at this time (Fig. 4, Supplementary Fig. 3B online).

Other metabolic pathways being affected (*FDR* < 0.05) by the lactation stage included 9 metabolic pathways that participate in lipid metabolism (Table 3; Fig. 3). These metabolic pathways were mostly related with greater concentrations of several lipids and metabolic intermediates for 21 vs. 180 DIM. These metabolites included myristic, palmitic, arachidonic acids , and glycerol, among others (Fig. 4; Supl. Figure 3D). Finally, metabolic pathways belonging to nitrogen metabolism as well as redox metabolism, and vitamins and coenzymes metabolism were also observed to be affected by DIM.

### Effect of genetic strain on metabolic pathways

Metabolic pathways differing (*FDR* < 0.05) between genetic strains were only detected at 21 DIM. The BCAA biosynthesis and degradation, and lysine degradation were shift-regulated (*FDR* = 0.022) between genetic strains as they were associated with greater concentrations of several metabolites for NAH vs. NZH cows (Table 3, Figs. 3 and 5). Differences in BCAA pathways were associated with greater concentrations (*FDR* < 0.05) of the three BCAA (valine, leucine, isoleucine) for NAH vs. NZH cows, as well as intermediary metabolites (e.g.: 2-ketoisocaproic, 2-ketobutyric) that were greater but did not significantly differ (Fig. 6). Lysine degradation was also linked with greater concentrations of L-pipecolic acid and $\alpha$-aminoadipic acid for NAH vs. NZH cows, but no significant differences were detected for lysine. In addition, seleno compounds metabolism and aminoacyl-tRNA biosynthesis tended to be affected (*FDR* < 0.07) when comparing genetic strains as most of its involved metabolites (16/19) were greater for NAH vs. NZH cows in early lactation (Figs. 3, 5).

## Discussion

Modulating effects of the genetic background on energy and lipid homeorhetic adaptations of dairy cows facing challenging conditions imposed by pasture-based dairy systems have been widely reported^10^. However, the role of genetic strain on AA metabolism in relationship with energy and lipid metabolism has not yet been reported. Our results demonstrated that homeorhetic changes of AA metabolism in early lactation are likely affected by the genetic strain associated with differences in energy metabolism. The greater milk yield and lower body reserves of NAH vs. NZH cows associated with a greater degree of insulin resistance and uncoupling of the somatotropic axis in NAH cows^8,9^, could be also linked with chronic activation of the mTOR pathway through increased BCAA degradation^20^ in the NAH cows. These results are further discussed around two main aspects: a) differential effects of the Holstein genetic strain (NAH vs. NZH) on AA and energy metabolism during early lactation, b) effects of early vs. mid-lactation (21 vs. 180 DIM) on AA, energy, and lipid metabolic changes in grazing cows from a systemic point of view.

Our results indicated that differential effects of the Holstein genetic strain (NAH vs. NZH) on energy and AA metabolism, were evident only in early lactation. This suggests that the effect of the genetic strain on metabolic adaptive responses is exacerbated in highly challenging conditions such as the onset of lactation. Differences were mostly related to plasma concentrations of AA or AA-related compounds as most of these metabolites were increased for NAH vs NZH cows in early lactation. Indeed, increased plasma BCAA could be related to the greater insulin resistance previously demonstrated for NAH vs NZH cows under grazing conditions^9^ (Fig. 6). Circulating BCAA, which are poorly catabolized in the liver, can act as signaling molecules sensing the nutritional state and activating cellular signaling cascades^21^. However, a relationship between insulin resistance and BCAA has been recently suggested in ruminants^22^, and the exact mechanisms still remain to be known. In this sense, it is likely that greater BCAA would have upregulated the mTOR activity^23^ in NAH cows during early lactation (Fig. 6). Despite upstream activation pathways of the mTOR differ between insulin and BCAA, insulin fails to stimulate the mTOR downstream cascade in the absence of AA^24^. Moreover, in humans, it has been proposed that high BCAA concentrations would indirectly impair insulin sensitivity through the chronic activation of the mTOR pathway leading to over-phosphorylation of the insulin receptor substrate 1^20,25^ which ultimately determines a decreased expression and activity of glucose transporter 4^26^. However, the linking mechanism between insulin resistance and BCAA is not completely understood and whether high concentrations of BCAA are a cause or a consequence of insulin resistance development still remains to be known.

Additionally, greater BCAA concentrations for NAH than NZH cows could also be the consequence of increased muscle protein mobilization in early lactation. Hypothetically increased activity of mTOR for NAH cows would enhance protein synthesis^27^. Therefore, it is possible that NAH cows had not only greater muscle mass breakdown but also protein synthesis, leading to increased protein turnover when compared with NZH cows. Indeed, Ghaffari et al.^18^ recently suggested that lower insulin sensitivity in obese dairy cows would be associated with increased protein turnover when compared to lean cows.

Plasma concentrations of alanine and $\alpha$-ketoisocaproic were greater for NAH than NZH cows suggesting increased catabolism of the BCAA in NAH animals^28^ (Holecek et al., 2018). It has been observed that low intracellular glucose concentrations, due to reduced glucose uptake by peripheral tissues associated with reduced insulin sensitivity^29^, would enhance BCAA degradation through the glucose-Krüppel-like factor 15 (KLF15)-BCAA axis^30^. These latter authors demonstrated that low glucose levels down-regulate the negative feedback of the KLF15 transcriptional regulator factor at the BCAA decarboxylation step which in turn is the limiting rate step of BCAA catabolism. In addition, alanine as well as the degradation products of BCAA replenish the oxalacetate pool or enter into the TCA cycle in order to produce glucose or energy, reflecting the interconnection between protein and energy metabolism^26,31^. Actually, a greater oxalacetate redirection from the TCA cycle towards gluconeogenesis would determine a down-regulation state of this cycle (cataplerosis) leading to the reduced citrate, aconitate and $\alpha$-ketoglutarate concentrations observed for NAH vs. NZH cows (Fig. 6) during early lactation^32,33^. In addition, the expected greater DMI of NAH cows (due to its greater BW, + 8%), in addition to increased valine catabolism leading to greater propionyl-CoA, could explain the increased plasma concentrations of succinate and fumarate observed for NAH vs. NZH cows^34^. Taken together our results point at a greater BCAA degradation rate and decreased TCA cycle activity for NAH vs. NZH cows.

Interestingly, other metabolites such as $\alpha$-aminoadipic, which were increased for NAH than NZH cows, are probably also related with greater insulin resistance. Indeed, $\alpha$-aminoadipic acid, a product of lysine degradation, has been previously reported as a pre-diabetic biomarker in rodents and humans^35^ as well as in dairy cows^18^. In addition, both, $\alpha$-aminoadipic acid and L-pipecolate, another metabolite of the lysine degradation, were reported to be elevated in blood samples of patients with liver injury and peroxisomal disorders in humans^36^. The greater concentrations of these metabolites observed for NAH vs. NZH cows would be in agreement with the reduced liver mitochondrial functionality observed by our team for NAH cows^37^.

Independently of their genetic strain, all cows showed several metabolic changes across lactation stages which confirmed several links widely reported between AA, and energy and lipid metabolism during negative energy balance in early lactation. The general trend for decreased plasma concentrations of AA in early lactation was in agreement with previous reports^38^ reflecting a state of negative protein balance at 21 DIM^39,40^ because of increased AA requirements for milk protein synthesis and gluconeogenesis together with a decreased dry matter intake after calving^39,41,42^. In contrast to the general trend for decreased plasma AA, the tendency of greater plasma glycine and greater concentrations of trans-4-hydroxyproline in early than mid-lactation suggest an increased muscle protein breakdown coupled with increased AA de novo synthesis at 21 vs. 180 DIM^38,43^. Indeed, the greater concentrations of trans-4-hydroxyproline in early lactation could reflect increased muscle protein breakdown at this time as its plasma concentrations would probably be indicative of connective tissue amounts of musculoskeletal system^44,45^.

In addition to decreased AA plasma concentrations, our results indicate that the urea cycle was down-regulated at early lactation probably linked with the increased demand for energy production^17^ in agreement with studies that reported a decreased activity of urea cycle enzymes around parturition^46,47^. Indeed, all measured compounds related to the urea cycle (e.g.: urea, ornithine, citrulline, aspartate), except fumarate, were lower for early than mid-lactation. In this sense, our results reinforce the hypothesis formulated by Kuhla et al.^43^ which states that the enhanced muscle breakdown during early lactation provides substrates for milk synthesis in parallel with a slowed-down activity of the urea cycle. Consequently, an enhanced nitrogen use efficiency would be expected to occur during early lactation, which is consistent with the lower plasma and milk concentrations of urea observed in our study^48,49^. In agreement with recent ly reported data by Luo et al.^17^, greater fumarate together with decreased glutamate, which can act as a shuttle between the urea and the TCA cycles, suggest that the slowed-down activity of the urea cycle could happen to sustain increased TCA activity through the synthesis of fumarate. Indeed, the metabolic pathway analysis indicated that the TCA cycle was enhanced in early vs. mid-lactation as previously reported for confined dairy cows^50^. The increased plasma concentrations of citrate and isocitrate agreed with increased activities of citrate synthase and isocitrate dehydrogenase previously reported during negative energy balance of early lactation^51,52^.

Moreover, greater TCA activity during early lactation is concordant with increased NEFA and saturated free fatty acids (*e.g.:* palmitic and stearic acids) concentrations due to adipose tissue mobilization after calving as they are main components of adipose tissue^53,54^. Additionally, greater concentrations of unsaturated fatty acids such as linoleic, linolenic, and arachidonic acids suggest that early lactation was associated with oxidative stress and a proinflammatory state^55^. In fact, polyunsaturated fatty acids (**PUFA**), which are well-known precursors for oxylipid synthesis^56^ through its metabolization by the cyclooxygenase/lipoxygenase pathway, provide a link between lipid mobilization, and oxidative stress and inflammation during the transition period^55,57^.

Lastly, decreased concentrations of ketogenic AA such as lysine, tyrosine, and phenylalanine in early lactation might have accounted for increased ketone body synthesis^58^, which is further depicted by increased plasma concentrations of BHB. Indeed, metabolic pathway analysis revealed that the butanoate metabolism was differentially expressed between stages of lactation. The decreased concentrations of $\alpha$-ketoglutarate in early lactation could be due, at least in part, to increased activity of 2-hydroxyglutarate dehydrogenase leading to the observed greater concentrations of 2-hydroxyglutarate^59^, which is further used in the synthesis of acetoacetyl-CoA. In turn, acetoacetyl-CoA is metabolized via acetoacetyl-CoA and $\beta$-hydroxy-$\beta$ -methylglutaryl-CoA (HMG-CoA) in the liver mitochondria^59^.

Regarding the overall effects of the lactation stage on energy and lipid metabolism, our results confirm data widely reported in both confined and pasture-based dairy systems. However, pasture-based dairy systems impose great variation in the quality and availability of pastures which determines often changes in AA intake along the year^60^; therefore, further research is needed to better know the homeorhetic interactions between AA metabolism, and energy and lipid metabolism when grazing dairy cows are subjected to changing nutritional environments.

## Conclusions

In agreement with reported data for confined systems, our results confirmed decreased plasma AA and increased fatty acid concentrations concomitant with a down-regulation of the urea cycle, and an up-regulation of the TCA cycle in early vs. mid-lactating grazing cows. However, more studies are needed for a better understanding of homeorhetic interactions between specific AA and energy metabolism in cows managed in pasture-based dairy systems. In addition, BCAA possibly act as signaling molecules behind differences in adaptive metabolic responses of NAH vs. NZH genetic strains when managed under grazing conditions.

## Methods

### Experimental design and management

The experiment was carried out at the Experimental Research Station “La Estanzuela” (34°20’ S, 57°40’ W) belonging to the Instituto Nacional de Investigación Agropecuaria (INIA) of Uruguay. This study was carried out in compliance with the ARRIVE guidelines and all procedures were approved by the Ethic Committee on Animal Experimentation of INIA (form INIA_2017.2).

Cows were randomly selected from a larger experiment designed to study the interaction between genetic strains and grazing-based feeding strategies previously reported by Talmon et al.^61^. Multiparous dairy cows ($3.1 \pm 0.9$ lactations; fall calving 5/15/$2018 \pm 12$ days, mean ± SD) of NAH (n = 8) and NZH (n = 8) genetic strains were used. Cows were fed a strategy that maximized pasture grazing. Previous to calving, cows had a LW of 593 ± 17 and 560 ± 17, and a BCS of 2.94 ± 0.06 and 3.28 ± 0.06 for NAH and NZH cows, respectively (mean ± SD). At least 87.5$\%$ of each cow’s ancestors (three generations) had an American (USA or Canada) or New Zealand proved origin for NAH and NZH genetics strains, respectively (Mejoramiento y Control Lechero Uruguayo; https://www.geneticalechera.com.uy/). The 305-days expected milk yield was 7500 and 5500 kg and the economic and productive selection index was 104 and 130 on average for NAH and NZH cows, respectively. The NAH cows had an expected progeny difference of +70.4 kg, +0.01 % fat and -0.01 % protein for milk yield, milk fat and milk protein content, respectively, when compared to the national herd. The NZH cows had an expected progeny difference of -185.7 kg, +0.23 % fat and +6.19 % protein for milk yield, milk fat and milk protein content, respectively, when compared to the national herd.

The feeding strategy was designed to maximize pasture grazing according to weekly pasture growing rate, and concentrate was offered twice a day individually (at a rate of 33% of the predicted daily dry matter intake) at the milking parlor. Cows were outdoors all year-round and throughout the experiment cows grazed orchard grass + lucerne (*Dactylis glomerata* + *Medicago sativa*, 75% of the grazing time) or fescue (*Festuca arundinacea*, 25% of the grazing time) on a rotational grazing system with strips assigned after milking and free access to freshwater. In order to avoid ingestion behavior interferences or dominance between genetic strains due to body size differences, each group was offered its own daily strip of pasture to keep a similar herbage allowance relative to their BW. Greater details on pasture and grazing management are available on Supplementary Table S1 online as well as in the work published by Talmon et al.^61^. Conserved forage was offered in a feeding parlor immediately before the afternoon milking. On average, the diet was comprised of 64% of grazed pastures, 5% of concentrate, and 31% of conserved forage (corn silage and pasture haylage mix; 73:27 ± 8% on dry matter basis, respectively). Further details of diet offered are available in Supplementary Table S1 online.

### Animal measurements and sample collection

Cows were milked twice a day at 0400 and 1400 h. Milk yield was recorded daily and weekly milk samples were collected for composition analysis (Combi FOSS FT+, Foss Electric, Hillerhød, Denmark). Cow body weight and BCS (scale from 1 to 5, Edmonson et al.^62^) were recorded once every two weeks. Fat and protein corrected milk (FPCM) yield was calculated according to Østergaard et al.^63^ Blood samples were collected at early and mid-lactation (21 and 180 ± 3 days in milk; DIM) (mean ± SE) by caudal venipuncture using 10 mL heparinized Vacutest ®tubes (Vacutest Kima, Arzergrande, Italia). Plasma samples were harvested by centrifugation (4000 $\times$ g, 12 min) and immediately stored at -80°C until analysis. Plasma glucose, NEFA, $\beta$-hydroxybutyrate (BHB), urea, and insulin concentrations were determined as previously reported^64^.

### Targeted metabolomic analysis and annotation

Metabolomic analysis and ion annotation procedures were performed by using gas chromatography time-of-flight mass spectrometry (GCToF/MS) according to Fiehn et al.^65^ at the core lab West Metabolomic Center (UC Davis Genome Center, Davis, CA, USA) and 200 metabolites were analyzed. A column of 30 m length $\times$ 0.25 mm internal diameter with 0.25 $\upmu$m film made of 95% dimethyl/5%diphenylpolysiloxane (Rtx-5Sil MS, Restek®Corporation,) was used for chromatography analyses. A Leco®Pegasus IV mass spectrometer (St Joseph, MI, USA) was used with unit mass resolution at 17 spectra/s from 80 to 500 Da and ionization energy set in -70 eV and equipped with an 1800 V detector voltage, 230 °C transfer line, and a 250°C ion source. Chromatographic data were pre-processed without smoothing and peak width was set at 3 s, the baseline subtraction was done just above the noise level, and automatic mass spectral deconvolution and peak detection at signal/noise levels of 5:1 were automatically performed throughout the chromatogram. Annotation of derivatized ions was performed using the BinBase algorithm based on deconvoluted spectra and peak metadata (retention index, unique ion, spectral similarity, signal/noise ratio, peak purity) from the LecoChromaTOF software using a multi-tiered filtering system with stringent thresholds, specifically developed for GCToF/MS data annotation^66^. Data is reported as peak height normalized by the sum of all peak heights (mTIC) of each sample as previously reported by Fiehn et al.^65^.

### Statistical analysis

Productive performance and metabolite and endocrine concentrations were analyzed as repeated measures using the MIXED procedure (SAS University Edition, SAS Institute Inc., Cary, NC, USA). The model included genetic strain, lactation stage (21 vs. 180 DIM) and their interaction as fixed effect, and cow as a random effect.

Metabolomic data pre-processing and statistical analysis were performed using MetaboAnalyst v4 (https://www.metaboanalyst.ca/). Prior to statistical analysis, data were normalized by the median, cube root transformed, and auto-scaled according to Chong et al.^67^. Data quality was assessed through multivariate analysis comparing individual samples’ data against the pooled samples. Data were subjected to multivariate analysis: clustering was assessed by PCA and classification models were assessed by PLS-DA^68^.

Data were also subjected to ANOVA analysis considering a time-series model in which genetic strain (NAH vs. NZH), lactation stage (21 vs 180 DIM), and its interaction were considered as fixed effects, while the cow was considered as a random effect. Raw-P values were adjusted for multiple hypothesis testing^69^ at a false discovery rate of 5 % (*FDR* $\le$ 0.05). The time-trend of metabolites was complementarily assessed both by ANOVA simultaneous component analysis (**ASCA**)^70^ and Bayes statistical time-series analysis (**MEBA**)^71^. The ASCA analysis was performed on the basis of a model for each fixed effect, this is genetic strain, lactation stage, and interaction models. Each model was validated according to a permutation test being the significance threshold *P* < 0.05. If a model was significant, then the metabolites were evaluated according to leverage and SPE. Therefore, a given metabolite was considered to be affected by the genetic strain in a time-dependent manner if it was well-modeled by the ASCA interaction model, this is when the metabolite had high leverage (> 0.04) and low SPE (< 1.5 $\times$ 10$^{-30}$) values. Additionally, MEBA was performed based on Hotteling’s T$^{2}$, which is a Student’s t-statistic generalization for multivariate analysis. It was used to rank the metabolites according to their difference in temporal profile when comparing genetic strain^71^. The higher position in the ranking, the higher differences in temporal profiles among genetic strains.

Finally, metabolic pathways analysis was based on the *Bos taurus* KEGG database, combining the Globaltest, which calculates the association between the metabolite sets and the phenotype without referring to a background^72^, and a topologic analysis based in the betweenness centrality, which is a measure of the importance of a compound within a given metabolic pathway^67^. Significant enrichment of metabolic pathway was set at *FDR* $\le$ 0.05, and only metabolic pathways with at least two metabolites quantified in the current data set were further considered for discussion purposes. The metabolic pathway analysis was performed based on three comparisons: (a) 21 vs. 180 DIM, (b) NAH vs. NZH at 21 DIM, and (c) NAH vs. NZH at 180 DIM.

## Supplementary information

Supplementary Figures.

Supplementary Tables.
