# Supplementary material for: Plasma concentrations of branched-chain amino acids differ with Holstein genetic strain in pasture-based dairy systems
Source: Sci Rep. 2021 Nov 17;11:22414. doi: 10.1038/s41598-021-01564-0 (PMC8599868; doi:10.1038/s41598-021-01564-0)
Supplement: Supplementary file 1 — Supplementary Figures. [file 41598_2021_1564_MOESM1_ESM.pdf]

Branched-chain amino acids are associated with Holstein genetic strains differences in metabolic adaptations to pasture-based dairy systems

Ezequiel Jorge-Smeding[1], Mariana Carriquiry[1], Gonzalo Cantalapiedra-Hijar[2], Alejandro Mendoza[3], Ana Laura Astessiano[1]

1: Universidad de la República, Facultad de Agronomía, Departamento de Producción Animal y Pasturas, Av. Garzón780, Montevideo, Uruguay
2: INRAE, Université Clermont Auvergne, UMRH, 63122, Saint-Genes-Champanelle, France
3: INIA, EE La Estanzuela, Programa Nacional de Producción de Leche, Ruta 50, km 11, Semillero, Uruguay

*Corresponding author: ejorgesmeding@gmail.com

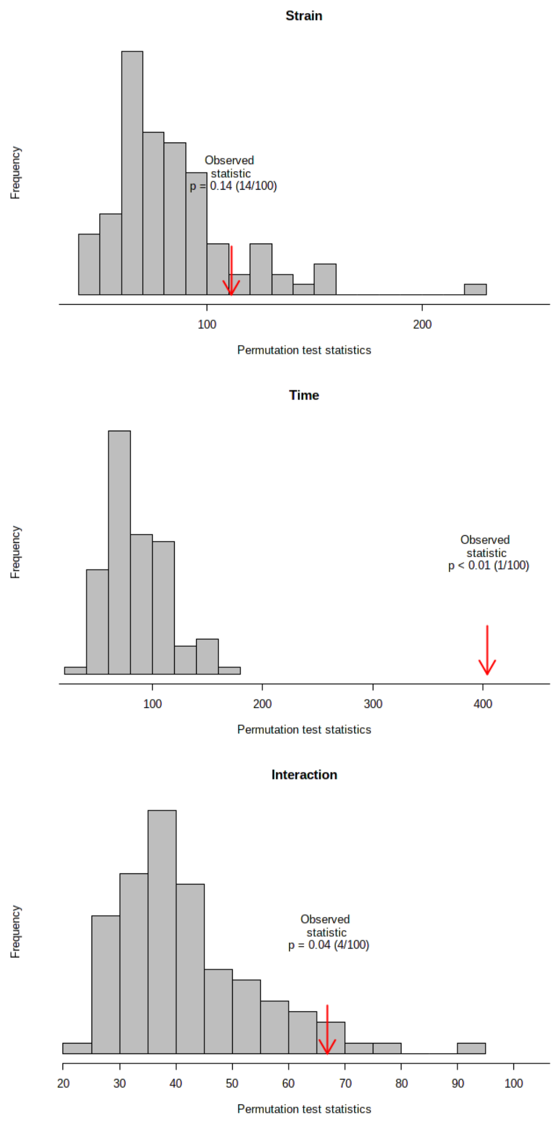

Supl. Figure 1. Validation statistics for fixed effects of ASCA analysis.

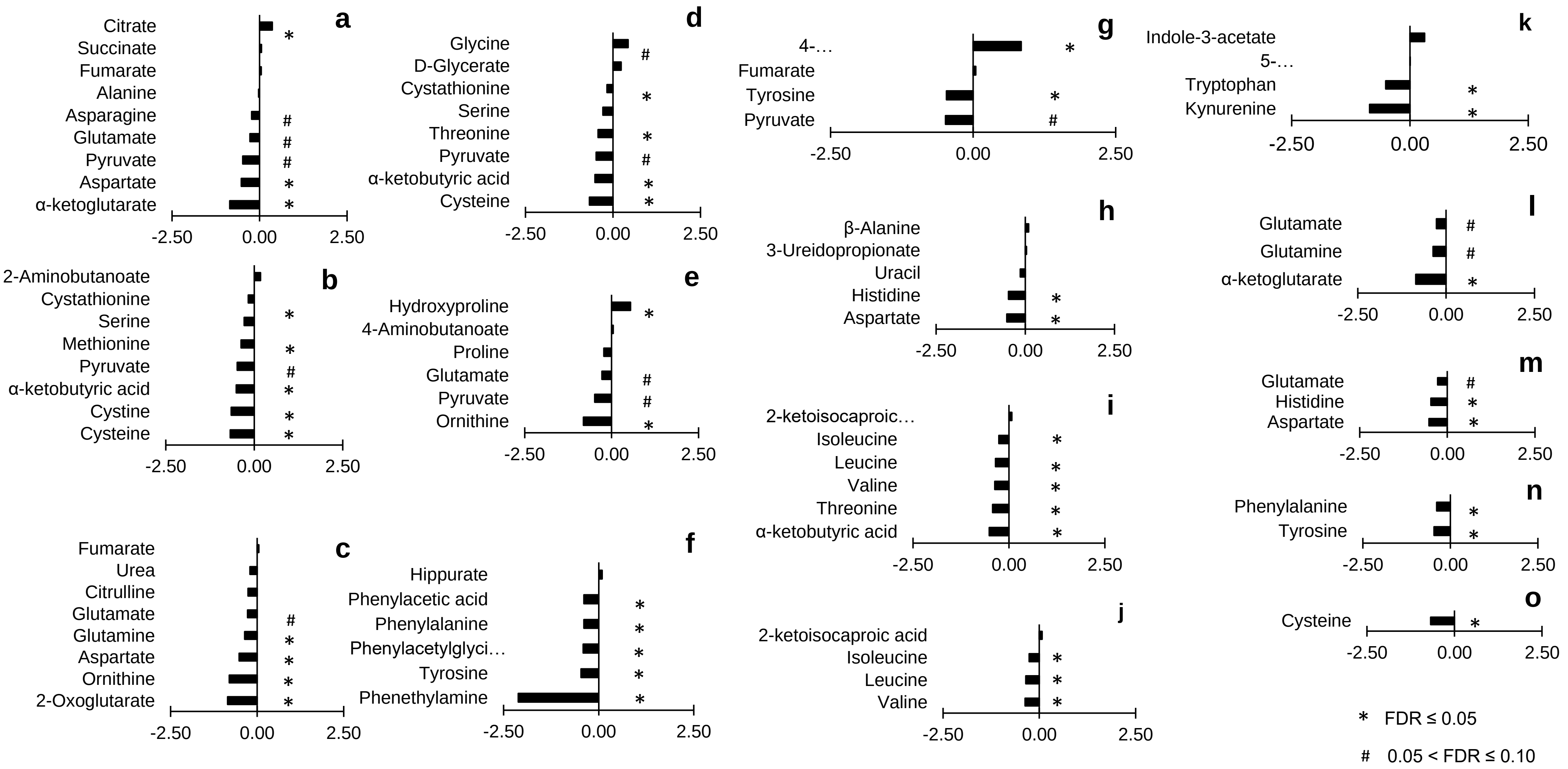

Supl. Figure 2. Log 2-fold change of measured metabolites belonging to AA and nitrogen metabolic pathways differing between 21 and 180 DIM: **a**) Alanine, aspartate and glutamate metabolism; **b**) Cysteine and methionine metabolism; **c**) Arginine biosynthesis; **d**) Glycine, serine and threonine metabolism; **e**) Arginine and proline metabolism; **f**) Phenylalanine metabolism; **g**) Tyrosine metabolism ; **h**) beta-Alanine metabolism ; **i**) Valine, leucine and isoleucine biosynthesis; **j**) Valine, leucine and isoleucine degradation; **k**) Tryptophan metabolism ; **l**) D-Glutamine and D-glutamate metabolism; **m**) Histidine metabolism; **n**) Phenylalanine, tyrosine and tryptophan biosynthesis; **o**) Thiamine metabolism, and taurine and hypotaurine metabolism.

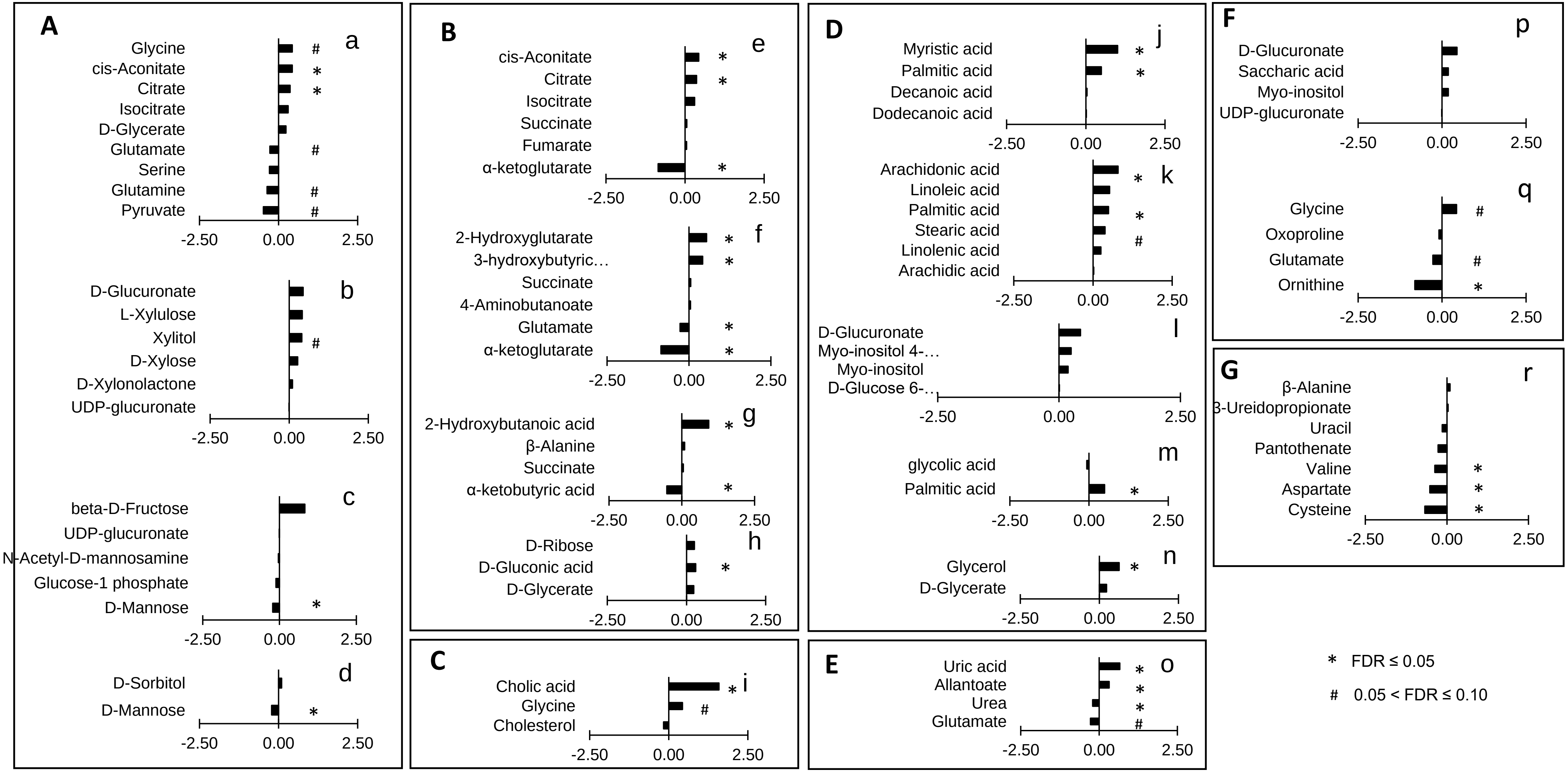

Supl Figure 3. Log 2 fold change of measured metabolites belonging to: **A**) carbohydrates metabolism [a) Glyoxylate and dicarboxylate metabolism; b) Pentose and glucuronate interconversions; c) Amino sugar and nucleotide sugar metabolism; d) Galactose, and fructose and mannose metabolism]; **B**) energy central metabolism [e) Citrate cycle; f) Butanoate metabolism; g) Propanoate metabolism; h) Pentose phosphate pathway]; **C**) Primary bile acids biosynthesis; **D**) Lipid metabolism [j) Fatty acid biosynthesis; k) Biosynthesis of unsaturated fatty acids; l) Inositol phosphate metabolism; m) Fatty acid degradation; n) Glycerolipid metabolism]; **E**) Nitrogenous bases metabolism [o) Purine metabolism]; **F**) Redox metabolism [p) Ascorbate and aldarate metabolism; q) Glutathione metabolism]; **G**) Vitamins and coenzymes [r) Pantothenate and CoA biosynthesis] .
